# Clinical Challenges in Resection and Reconstruction of a Verrucous Carcinoma of the Right Calcaneus With a Medial Plantar Artery Perforator Flap: A Case Report

**DOI:** 10.7759/cureus.13118

**Published:** 2021-02-04

**Authors:** Frank De Jongh, Sjaak Pouwels, Andreas Marinelli, Wouter F Willems

**Affiliations:** 1 Plastic Surgery, Haaglanden Medisch Centrum, The Hague, NLD; 2 Intensive Care Medicine, Elisabeth-Tweesteden Hospital, Tilburg, NLD; 3 Surgery, Haaglanden Medisch Centrum, The Hague, NLD

**Keywords:** verrucous carcinoma, carcinoma cuniculatum, dermatology, plastic surgery, medial plantar artery perforator flap

## Abstract

A verrucous carcinoma is a rare, low-grade variant of a well-differentiated squamous cell carcinoma (SCC). It frequently occurs in Caucasian males aged 50 to 60. The tumour is locally destructive, grows into muscle, nerves and bones, but rarely metastasizes. Here we report a patient with verrucous carcinoma on the right calcaneus with the uncommon symptom of a haemorrhagic plaque at the centre and also an exophytic component. A 52-year-old man presented with a 10-year-old, progressive, painful, pruritic, exophytic growing, hyperkeratotic and haemorrhagic plaque of 5.2 x 3.5cm on the right calcaneus. The lesion emerged after extensive burns after blast trauma as a child. Excisional biopsy with 2mm margin in combination with clinical presentation favoured a verrucous carcinoma. The pathology report showed that the tumour was not excised radically; therefore, re-excision with 5mm margin was required and was performed two months later. The wound was closed with a medial plantar artery perforator (MPAP) flap. The donor site was closed with a full-thickness skin graft (FTSG) from the medial side of the right upper leg and was covered by a tie-over bandage. Verrucous carcinoma is a rare tumour and can be adequately surgically treated by excision with clear margins. In this case, the verrucous carcinoma of the right calcaneus was excised and reconstructed with an MPAP flap with decent results, despite a challenging postoperative course.

## Introduction

Verrucous carcinoma is a rare, low-grade variant of a well-differentiated squamous cell carcinoma (SCC) that frequently occurs in Caucasian males aged 50 to 60. While this tumour is locally destructive and grows into muscle, nerves and bones, it rarely metastasizes. Survival rates are high, depending on the location [1-3]. Histopathological characteristics typically include a mixed endo- and exophytic growth pattern, keratin-filled crypts, keratin cores, and a relative lack of cytological atypia and malignant features [1-4]. It was first found on the plantar aspect of the foot, but has also consistently been described in other areas of the skin and mucosa, such as the mouth and anal region [5].

Here we report a patient with verrucous carcinoma on the right calcaneus with the uncommon symptom of a haemorrhagic plaque at the centre and also an exophytic component. Moreover, we report the challenges postoperatively (after resection and plastic surgical reconstruction).

## Case presentation

**Figure 1:**
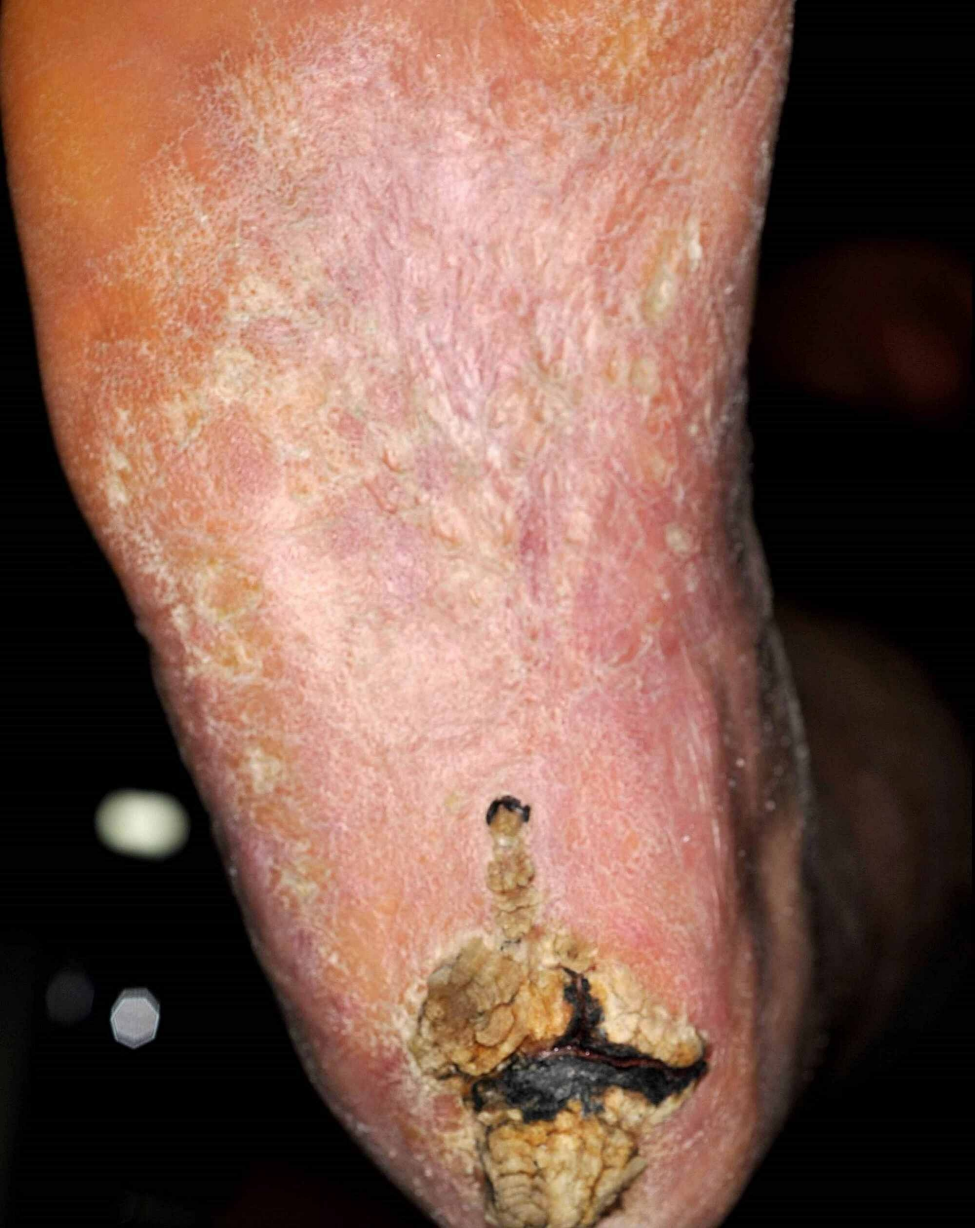
Verrucous carcinoma with a central growing hyperkeratotic haemorrhagic plaque of the right calcaneus

**Figure 2:**
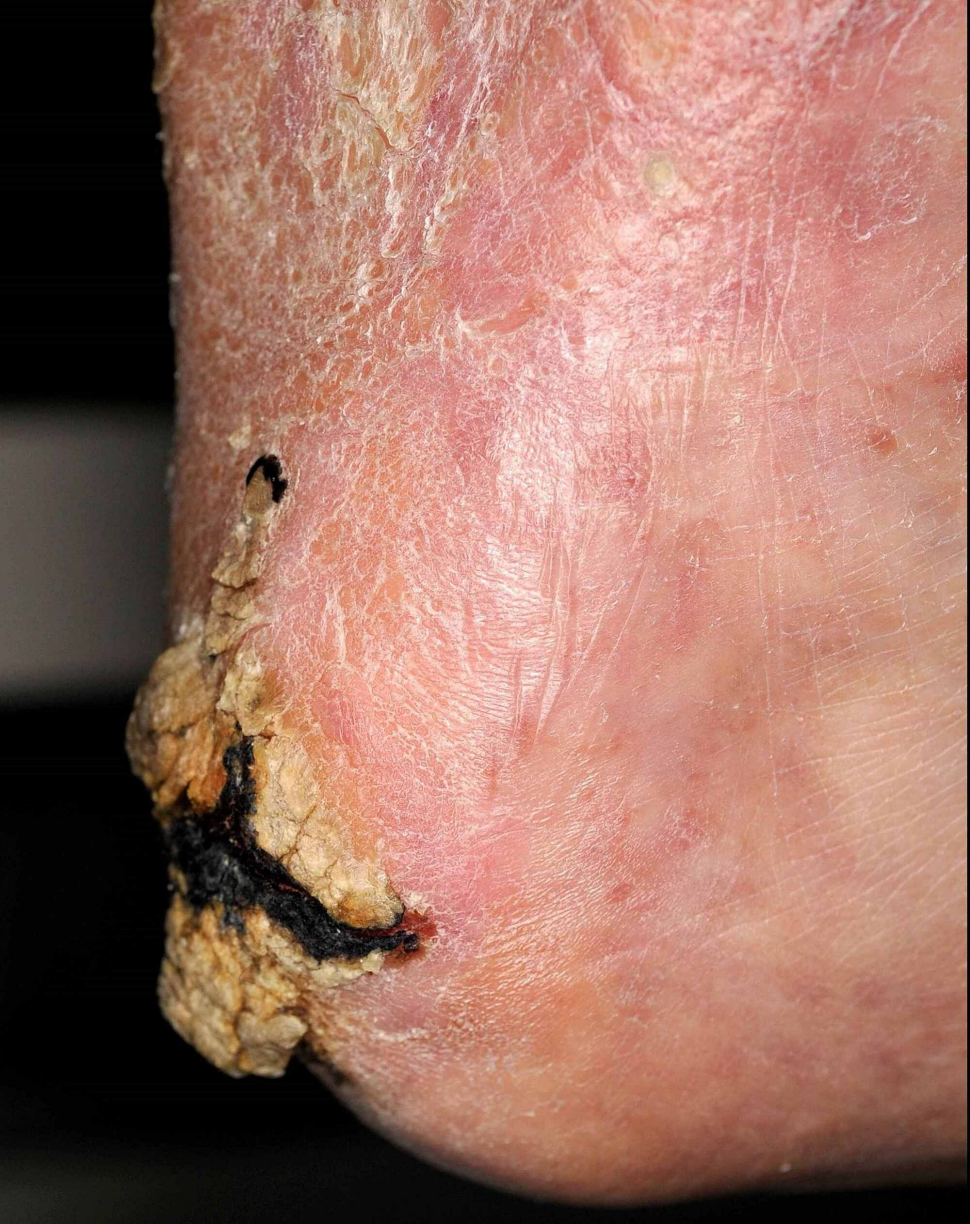
Medial side view of a verrucous carcinoma of the right calcaneus

A 52-year-old man presented with a 10-year-old, progressive, painful, pruritic, exophytic growing, hyperkeratotic, haemorrhagic plaque of 5.2 x 3.5cm on the right calcaneus (Figures 1-2). The lesion emerged after extensive burns following a blast trauma during childhood. The patient had a significant medical history of intellectual disability, left-sided transtibial amputation (as a result of a non-healing burn ulcer) and cholesteatoma. Excisional biopsy with 2mm margin in combination with clinical presentation favoured a verrucous carcinoma. An ultrasound showed a superficial tumour. No further diagnostic tests were done to assess lymph node status, due to the explicit wishes of the patient. The wound was closed ad secundam. The pathology report showed that the tumour was not excised radically; therefore re-excision with 5mm margin was required and was performed two months later. The wound was closed with a medial plantar artery perforator (MPAP) flap. The donor site was closed with a full-thickness skin graft (FTSG) from the medial side of the right upper leg and was covered by a tie-over bandage. After the operation, the leg was kept in an elevated position.

**Figure 3:**
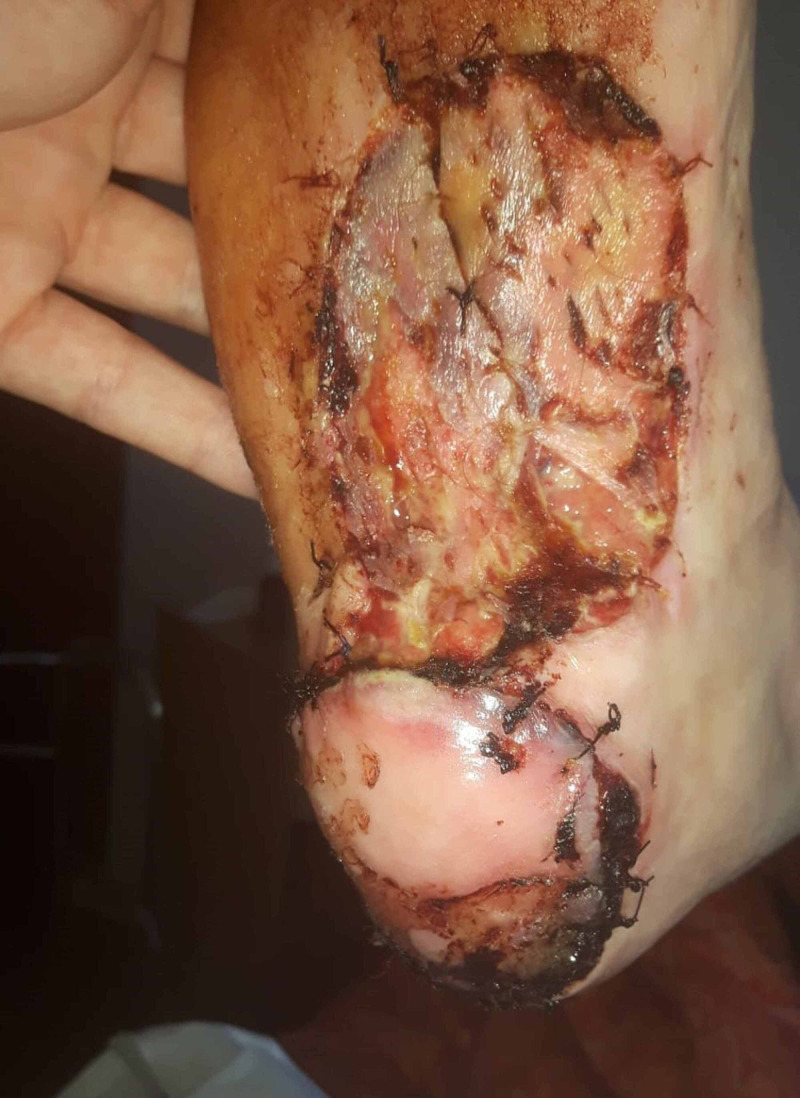
Result after surgical debridement of necrotic tissue of the MPAP-flap and the FTSG MPAP: medial plantar artery perforator; FTSG: full-thickness skin graft

**Figure 4:**
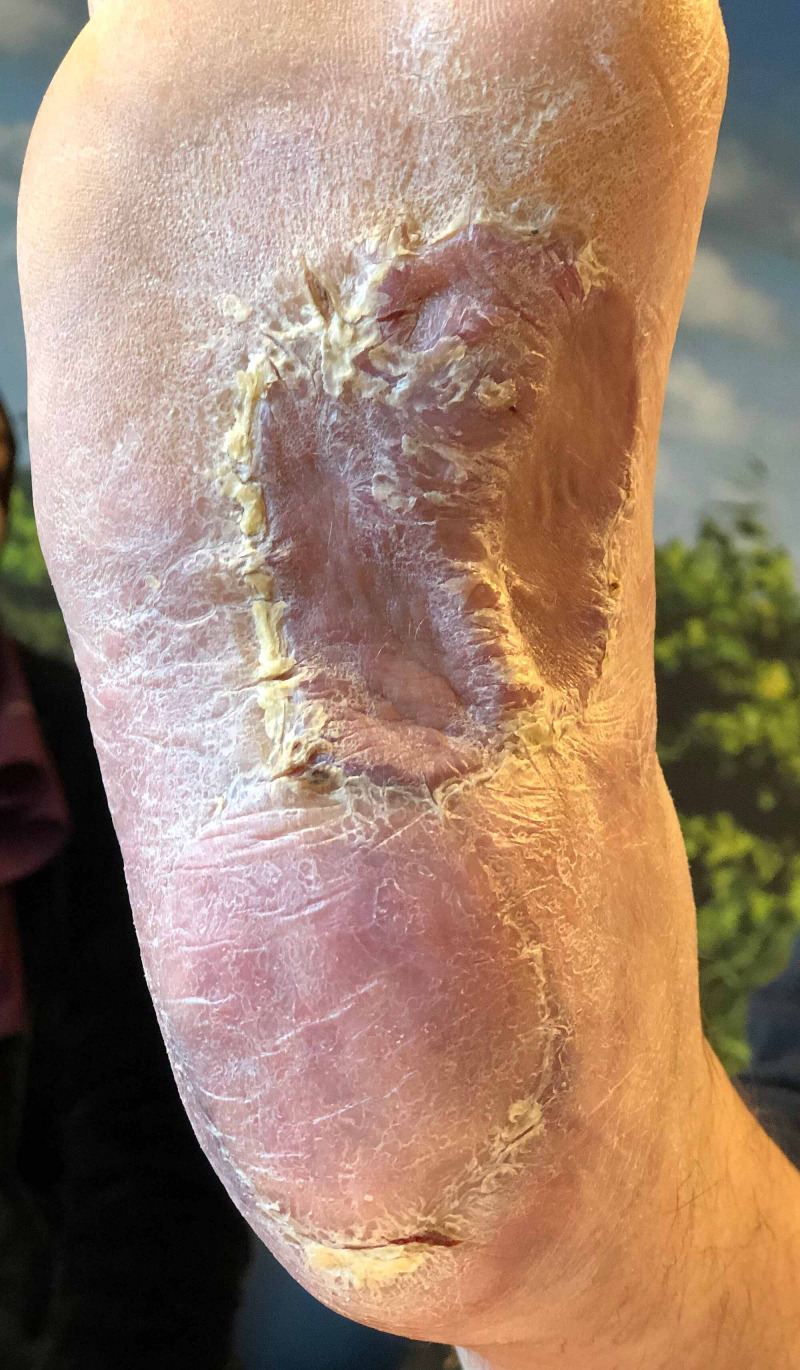
Fully healed MPAP-flap and FTSG, six weeks after surgery MPAP: medial plantar artery perforator; FTSG: full-thickness skin graft

Two days post-operatively, the MPAP-flap showed venous congestion at the distal periphery. Locally, the epidermis was excised and covered with heparin gauze to improve venous drainage for five days, after which normal vascularization recurred. At the lateral side of the inner foot (donor site of the MPAP flap), the FTSG only had partial take and was treated with Betadine gauze (Figure 3). Six weeks after surgery, all wounds were healed (Figure 4). The patient was allowed to walk freely after 10 weeks.

## Discussion

Our report describes a patient with verrucous carcinoma of the right calcaneus with an exophytic component and rare component of haemorrhagic plaque in the centre. Verrucous carcinomas are a rare variant of squamous cell carcinoma (SCC). These carcinomas are slow-growing with low metastatic potential and the clinical presentation is variable [6]. The growth pattern of these lesions often incorporates an exophytic pattern and initial presentation is often as an ulcus. [6, 7]. The inverted variant of verrucous carcinoma (carcinoma cuniculatum) has some distinct histopathological features. Mainly the extensive crypts and ramifying sinuses are histopathologically unique for this type of verrucous carcinoma [8]. Often, keratotic debris is seen within the crypts and sinuses, which are lined with well-differentiated keratinized, stratified, squamous epithelium with hyper- and parakeratosis and a prominent stratum granulosum [6, 9, 10]. Despite the absence of cellular atypia and normal mitotic figures [6], bony invasion has been reported in several cases [1, 11]. Thus, these lesions may display relatively benign histology with concomitant locally invasive behaviour [10].

Regarding the pathophysiology of verrucous carcinoma, not much is known. Several theories have been proposed; human papillomavirus (HPV), chronic inflammation/infection and trauma [1, 12]. In cases of verrucous carcinomas of the oro-aerodigestive or ano-urogenital tract, there has been no clear association with HPV. Some of them show an association with low- and high-risk strains of HPV (respectively, 2% to 12% and 5% to 24%) [13-15]. Despite some evidence, the role of HPV in the pathogenesis of verrucous carcinomas is still not fully understood and immunohistochemical and electron microscopy evidence is lacking [8, 16, 17].

Because of the occurrence in weight-bearing areas like the plantar, foot and sacrogluteal region, pressure has also been raised as a potential causative factor [8, 10]. This might be caused by obstruction and possible secondary inflammatory changes. Another pathophysiological explanation could be exposure to (tissue) trauma and toxins in these more vulnerable areas of the human body (good examples include oropharyngeal mucosa, feet, and hands) [7, 10]. Despite possible multifactorial pathophysiology, verrucous carcinomas tend to occur mostly in males between 50 and 60 years [1]. General recommended treatment is wide local excision with a recommended 4mm to 5mm margin [10].

Reconstruction of soft-tissue defects of the calcaneus can be challenging, which was also the case in our patient. In the calcaneus region, reconstruction needs to be done with tissue that can resist the body weight. In other words, the reconstruction of these body weight-bearing tissues is a unique assignment. A good option could be local tissue flaps. [18]. Reverse sural artery flaps [19], medial plantar artery perforator (MPAP) flap, or peroneal perforator artery flaps [20] are viable local options. Of course, each procedure and each flap has its pros and cons [20]. MPAP flaps are especially suitable for these reconstructions because of the use of thick, glabrous skin and its sensory feedback [18]. Besides the hind foot, this flap can also be a good option for the reconstruction of mid- and fore foot defect [18-20]. Several aspects make the use of MPAP flaps difficult: (1) difficulty of dissection, and (2) the long learning curve to fully master this surgical technique [18-20]. One of the advantages over, for example, the reverse sural artery flap, is that the MPAP flap gives sensory feedback due to the sensate skin fulfilling local demands [18, 19]. Unfortunately, tissue edema is a significant problem arising with both flaps (MPAP and reverse sural artery flap) [18-20]. This is probably due to the venous flow alterations and stasis of fluids [18]. This was also the case in our patient, who required additional treatment as described above, six weeks postoperatively.

## Conclusions

Verrucous carcinoma is a rare tumour and can be adequately surgically treated by excision with clear margins. In our case the verrucous carcinoma of the right calcaneus was excised and reconstructed with a medial plantar artery perforator flap with decent results, despite a challenging postoperative course.
